# Rheumatoid Arthritis Impacts on the Independent Relationships between Circulating Adiponectin Concentrations and Cardiovascular Metabolic Risk

**DOI:** 10.1155/2013/461849

**Published:** 2013-04-03

**Authors:** Patrick H. Dessein, Gavin R. Norton, Margaret Badenhorst, Angela J. Woodiwiss, Ahmed Solomon

**Affiliations:** ^1^Cardiovascular Pathophysiology and Genomics Research Unit, School of Physiology, Faculty of Health Sciences, University of the Witwatersrand, 7 York Road, Parktown, P.O. Box 1012, Melville 2109, Johannesburg 2193, South Africa; ^2^Department of Rheumatology, Charlotte Maxeke Johannesburg Academic Hospital, Faculty of Health Sciences, University of the Witwatersrand, 7 York Road, Parktown, Johannesburg 2193, South Africa

## Abstract

Adiponectin and leptin are likely involved in the pathophysiology of rheumatoid arthritis (RA) and therefore potential new therapeutic targets. Adiponectin inhibition could be expected to enhance cardiovascular metabolic risk. However, it is unknown whether RA changes the influence of adipokines on cardiovascular metabolic risk. We determined whether RA impacts on the independent relationships of circulating leptin and adiponectin concentrations with cardiovascular risk factors and carotid intima-media thickness (cIMT) in 277 black African subjects from a developing population; 119 had RA. RA impacted on the relationships of adiponectin concentrations with lipid concentrations and blood pressure, independent of confounders including adiposity (interaction *P* < 0.05). This translated into an association of adiponectin concentrations with more favorable lipid variables including HDL cholesterol (*P* = 0.0005), non-HDL cholesterol (*P* = 0.007), and triglyceride (*P* = 0.005) concentrations, total cholesterol-HDL cholesterol (*P* = 0.0002) and triglycerides-HDL cholesterol (*P* = 0.0003) ratios, and higher systolic (*P* = 0.0006), diastolic (*P* = 0.0004), and mean blood pressure (*P* = 0.0007) in RA but not non-RA subjects. Leptin was not associated with metabolic risk after adjustment for adiposity. The cIMT did not differ by RA status, and adipokine concentrations were unrelated to atherosclerosis. This study suggests that leptin and adiponectin inhibition may not alter overall cardiovascular risk and disease in RA.

## 1. Introduction

Since the identification of leptin in 1994, adipose tissue is no longer regarded as a passive reservoir for energy storage [1]. Rather, it comprises a highly active metabolic and endocrine organ that produces a large number of bioactive peptides [1–4]. These molecules are referred to as adipokines and substantially modulate metabolic cardiovascular risk factors including insulin resistance and atherogenesis as well as inflammatory and immune responses [5–9]. Leptin and adiponectin are the most studied adipokines. Leptin is primarily involved in food intake and energy expenditure but is also proinflammatory [1, 2]. Leptin production is increased in obesity and hyperleptinemia that reflects leptin resistance enhances metabolic risk [6]. By contrast, adiponectin is anti-inflammatory and improves metabolic risk, and its production decreases with increasing adiposity [1, 2, 5, 7]. 

 Investigations in patients with RA have mostly shown increased serum leptin and adiponectin concentrations, and both adipokines are also produced in inflamed joints [1, 10–19]. These findings support their involvement in the immune response in RA. Indeed, leptin induces interleukin-8 in RA synovial fibroblasts [20] but also has anabolic effects through stimulation of the expression of cartilage growth factors [1, 10]. In contrast to its anti-inflammatory role in the metabolic syndrome, adiponectin induces gene expression and protein synthesis in several effector cells involved in the pathophysiology of RA that result in the production of an extensive range of proinflammatory and prodestructive molecules [1, 10, 21–23]. Accordingly, leptin and adiponectin were proposed as potential new therapeutic targets in RA [23, 24]. However, whereas rheumatoid arthritis (RA) is associated with markedly enhanced metabolic cardiovascular risk and disease [25–27], inhibition of adiponectin would be expected to further enhance metabolic risk [5, 7].

 Available data on the potential contribution of adipokine metabolism to enhanced CVD in RA is mostly derived in white subjects from developed populations. Overall, serum adipokine concentrations as well as adipokine polymorphisms were found to be unrelated to atherosclerosis and cardiovascular event rates in RA [18, 28–31]. Importantly in the present context, the presence of autoimmunity can alter the impact of adipokines on cardiovascular metabolic risk and disease [32, 33]. Indeed, in a recent investigation by Hahn and colleagues, twice weekly intraperitoneally administered leptin increased proinflammatory high density lipoprotein scores and atherosclerosis in high fat diet fed lupus prone mice but not in nonautoimmune controls [32]. The same group also reported independent relationships between serum leptin and proinflammatory lipid concentrations as well as atherosclerosis in patients with lupus [33]. Of additional importance in the present context, differences between black and white subjects in the gene encoding adiponectin allele's frequencies as well as body types and metabolic risk factors according to ADPC genotypes were reported [34]. Whether RA modifies the influence of adipokines on metabolic risk, and atherosclerosis is currently unknown. In order to elucidate the potential anticipated effects of leptin and adiponectin inhibition on metabolic cardiovascular risk in RA, in the present investigation, we examined the impact of RA on the independent relationships of the respective adipokines with metabolic cardiovascular risk factors and carotid intima-media thickness (cIMT) in black African subjects that form part of a developing population.

## 2. Methods

### 2.1. Patients

The present investigation was conducted according to the principles outlined in the Helsinki declaration. The Committee for Research on Human Subjects of the University of Witwatersrand approved the protocols (approval numbers: M02-04-72 and renewed as M07-04-69 in non-RA subjects and M06-07-33 in RA subjects). Participants gave informed, written consent. The present study design has previously been described [35–42]. Briefly, 119 African black patients that met the 1988 American College of Rheumatology criteria for RA [43] were enrolled at the Charlotte Maxeke Johannesburg Academic Hospital and Milpark Hospital [35–38]. All invited patients agreed to participate. All patients used disease modifying agents for rheumatic disease (DMARD) at the time of the study. Age and sex matched non-RA subjects (*n* = 158) were participants in a population study on cardiovascular risk and disease that is also conducted in Johannesburg [39–42]. This investigation comprises randomly recruited nuclear families of black African descent with siblings older than 16 years. Serum leptin concentrations were measured in all non-RA and 112 of non-RA subjects and those of adiponectin in all RA and 77 of the non-RA subjects. Serum C-reactive protein (CRP) concentrations and carotid intima-media thickness (cIMT) were determined in 123 and 91 of the non-RA subjects. The other recorded variables did not differ in non-RA subjects with and without adiponectin, CRP, and carotid ultrasound assessments. Apart from the latter three investigations, data were missing in fewer than 5% for any of the recorded characteristics in all participants.

### 2.2. Baseline Characteristics

We recorded demographic features, life style factors comprising alcohol use (at least one unit per month), and exercise (at least once per month) that included time spent in walking, for example, to reach public transportation, cardiovascular, and nonsteroidal anti-inflammatory drug (NSAID) use. Height, weight, and waist and hip circumference were measured using standard approaches. Abdominal obesity and fat distribution were estimated by waist circumference and waist-hip ratio, respectively. CRP concentrations were determined using immunoturbidimetric methods. In patients with RA, we additionally recorded disease duration, the Clinical Disease Activity Index (CDAI) [44], rheumatoid factor status, and the use of traditional or synthetic DMARD. None of the patients were treated with biological DMARD therapy at the time of the study.

### 2.3. Metabolic Cardiovascular Risk Factors

Hypertension was defined as an average systolic blood pressure ≥140 or/and diastolic blood pressure ≥90 mmHg or/and current use of antihypertensive medications. Standard laboratory blood tests of renal and liver function, hematological parameters, lipids, and glucose were performed. Dyslipidemia was diagnosed when the atherogenic index; that is, the cholesterol-HDL cholesterol ratio was >4, and proatherogenic non-HDL cholesterol concentrations were calculated by subtracting HDL cholesterol from total cholesterol concentrations [35–38, 45]. We documented smoking habits. Diabetes was identified as the use of glucose lowering agents or a fasting plasma glucose ≥7 mmol/L. 

### 2.4. Carotid Artery Atherosclerosis

Carotid artery intima-media thickness (cIMT) measurements were made using a linear array 7.5 MHz probe attached to a high resolution B-mode ultrasound machine (SonoCalc IMT, Sonosite Inc, Bothell, Wash, USA) in both RA and non-RA subjects, as recently described [35, 37, 38]. This equipment involves the application of a unique semiautomated border detection program that was previously documented to provide highly reproducible intra- and interrater results in other as well as our settings [35, 37, 38, 46]. Carotid artery plaque is currently identified in our RA patients [35, 37, 38] but not in non-RA subjects, and hence results on plaque are not shown in the present report.

### 2.5. Leptin and Adiponectin Concentrations

Leptin and adiponectin concentrations were measured using solid-phase sandwich enzyme-linked immunosorbent assays (ELISA) (QuantakineHS, R&D Systems, Inc., Minneapolis, MN, USA). The lower detection limit was 7.8 pg/mL for leptin and 0.246 ng/mL for adiponectin. The inter- and intraassay coefficients of variation were 4.4 and 3.2%, respectively for leptin and 6.5 and 3.5%, respectively, for adiponectin. 

### 2.6. Data Analysis

Dichotomous variables are expressed as proportions or percentages and continuous variables as mean (SD). Nonnormally distributed characteristics were logarithmically transformed prior to statistical analysis and for these variables geometric means (SD) are given. 

 Disparities in baseline characteristics between RA and non-RA subjects were compared using the Students *t*-test and univariate logistic regression analysis as appropriate. Associations of RA with metabolic cardiovascular risk factors and cIMT were investigated in multivariable logistic and continuous regression models with consistent adjustment for demographic characteristics since age differed numerically by RA status and for antihypertensive, statin, and glucose lowering therapy as appropriate. 

 The relationships of RA with serum adipokine concentrations were first assessed by the Students *t*-test and subsequently in confounder adjusted multivariable linear regression models.

 The associations between baseline characteristics and adipokine concentrations and of adipokine concentrations with metabolic cardiovascular risk factors and cIMT were investigated in confounder adjusted linear regression models. The impact of RA on these relationships was determined by the addition of interaction terms (RA × variable of interest) and their individual terms to the models and in stratified analysis, that is, in RA and non-RA subjects separately. 

 Statistical computations were made using the GB Stat program (Dynamic Microsystems, Inc, Silver Spring, MD, USA) and SAS software, version 9.1 (The SAS Institute, Cary, NC).

## 3. Results

### 3.1. Baseline Characteristics in Subjects with and without RA

Table 1 shows the baseline characteristics in the study participants. Non-RA subjects consumed alcohol more frequently, exercised more extensively, and smoked more cigarettes per day than patients with RA. Except for waist-hip ratio, adiposity indices were reduced in RA compared to non-RA participants. Patients with RA employed antihypertensives more often and in larger numbers. None of the non-RA subjects used statins. Serum C-reactive protein concentrations were similar in RA and non-RA subjects. Mean (SD) disease duration and CDAI were 12.8 (9.2) years and 8.1 (1.6), respectively, in patients with RA; 17.6% experienced clinical RA remission (CDAI < 2.8) [44] at the time of the study. Seventy-seven percent tested rheumatoid factor positive. Methotrexate, chloroquine, sulphasalazine, leflunomide, azathioprine, tetracycline, cyclophosphamide, penicillamine, and prednisone were employed by 91.1, 79.8, 24.4, 20.2, 16.8, 10.1, 5.6, 4.2, and 1.7% of patients, respectively; none were using biological agents.

### 3.2. Metabolic Cardiovascular Risk Factor Profiles and Carotid Atherosclerosis in Subjects with and without RA

Table 2 gives the metabolic cardiovascular risk factors and atherosclerosis results in subjects with and without RA. The prevalence of hypertension, diabetes, blood pressure values, and glucose concentrations did not differ significantly between participants with and without RA. By contrast, lipid characteristics were consistently more favorable in subjects with RA compared to those without RA. The cIMT did not differ by RA status.

### 3.3. Serum Leptin and Adiponectin Concentrations in Subjects with and without RA

The geometric mean (SD) circulating leptin and adiponectin concentrations were 28 330.88 (2.75), 12 114.63 (2.35) pg/mL, 11.25 (1.83), and 7.28 (2.05) ng/mL in non-RA and RA participants, respectively (*P* < 0.0001 for both). 

 In all participants, eight of the baseline characteristics (Table 1) were associated with leptin or/and adiponectin concentrations: in age adjusted analysis, female sex related to leptin and adiponectin concentrations (partial *R* = 0.405 (*P* < 0.0001) and 0.141 (*P* = 0.049), resp.); in age and sex adjusted analysis, BMI, waist, angiotensin converting inhibitor, and statin use were associated with leptin concentrations (partial *P* = 0.634 (*P* < 0.0001), partial *R* = 0.559 (*P* < 0.0001), partial *R* = −0.126 (*P* = 0.04), and partial *R* = −0.158 (*P* = 0.009), resp.), and BMI, waist, waist-hip ratio, CRP concentrations and angiotensin converting enzyme inhibitor, diuretic, and statin use were associated with adiponectin concentrations (partial *R* = −0.142, *P* = 0.05), partial *R* = −0.284 (*P* < 0.0001), partial *R* = −0.182 (*P* = 0.01), partial *R* = −0.224 (*P* = 0.005), (partial *R* = −0.254 (*P* < 0.0001), partial *R* = −0.185 (*P* = 0.01), and partial *R* = −0.164 (*P* = 0.02), resp.).

 When age as well as the potential confounders of sex, BMI (for leptin), waist (for adiponectin), CRP concentrations and angiotensin converting inhibitor, diuretic, and statin use were adjusted for, leptin and adiponectin concentrations remained higher in non-RA compared to RA subjects (*P* < 0.0001 and 0.0002, resp.).

### 3.4. The Impact of RA on the Relationships between Serum Adipokine Concentrations, Metabolic Risk Factors and Carotid Atherosclerosis

Table 3 gives the age, sex, antihypertensive agent, statin, and glucose lowering drug use (as appropriate) adjusted associations of adipokine concentrations with metabolic cardiovascular risk factors and cIMT. RA impacted on the relationships between leptin concentrations and blood pressure, lipid and glucose concentrations and cIMT. These results translated into an association of leptin concentrations with high diastolic and mean blood pressure in RA but not non-RA subjects and a relationship of leptin concentrations with a high total cholesterol-HDL cholesterol ratio, triglyceride concentrations and the triglycerides-HDL cholesterol ratios in non-RA but not non-RA participants. RA impacted on the relationships between adiponectin concentrations, and blood pressure and lipid concentrations. These results translated into an association of adiponectin concentrations with high systolic and mean blood pressure as well as low LDL and high HDL cholesterol concentrations and low total-HDL cholesterol and triglycerides-HDL cholesterol ratios in RA but not non-RA subjects, and adiponectin concentrations were more strongly associated with non-HDL cholesterol concentrations and the triglycerides-HDL cholesterol ratio in RA compared to non-RA subjects. 

 We repeated the analyses in Table 3 with further adjustment for adiposity indices as well as other potential confounders (see above). As shown in Table 4, the associations of leptin concentrations with metabolic risk factors were no longer significant in either RA or non-RA subjects. However, adiponectin concentrations remained strongly associated with high systolic, diastolic, and mean blood pressure as well as low LDL and non-HDL cholesterol and triglyceride concentrations, high HDL cholesterol concentrations, and low total-HDL cholesterol and triglycerides-HDL cholesterol ratios in RA, whereas no associations with metabolic risk factors were present any longer in non-RA subjects. Adiponectin concentrations remained unassociated with cIMT in both RA and non-RA participants. The lack of significance for the associations of adipokine concentrations with triglyceride concentrations and the triglycerides-HDL cholesterol ratio despite the presence of relatively large partial correlation coefficients in non-RA subjects was due to the presence of large standard errors of the regression coefficients in the respective models (>0.135 compared to <0.096 in RA and non-RA subjects, resp.). 

 We reevaluated the associations of adiponectin concentrations with high blood pressure values in RA (Table 4) in additional models. This revealed that when other potential confounders including life style factors (smoking, exercise status, and alcohol use) and leflunomide use [47] were additionally adjusted for, the respective relationships were unaltered (partial *R* = 0.347, 0.270, and 0.329 and *P* = 0.0004, 0.007, and 0.0008 for systolic, diastolic, and mean blood pressure, resp.).

## 4. Discussion

In the present study performed in black African people from a developing population, comprehensive cardiovascular risk factor assessment in both persons with and without RA allowed us to compare the relationships of circulating adipokine concentrations with metabolic cardiovascular risk factors and cIMT between both groups. The most novel finding produced by this investigation is that RA impacts consistently on several potentially important independent adiponectin concentration-metabolic cardiovascular risk factor associations that translate into disparities in the respective relationships in RA compared to non-RA subjects. Leptin concentrations were not independently related to cardiovascular risk. Neither leptin nor adiponectin concentrations were associated with atherosclerosis. Leptin antagonism reduced disease severity in a preclinical animal model of rheumatoid arthritis [24], and several studies have indicated that adiponectin is involved in the progression of RA [1, 10, 21–23, 48, 49]. Therefore, our findings have important potential implications in the management of RA as related to the possible use of leptin and adiponectin inhibition in RA [23, 24].

 The absence of independent relationships of leptin concentrations in both non RA and RA subjects and adiponectin concentrations in non-RA subjects with metabolic risk, as found in our study, suggests that leptin and adiponectin are markers of fat mass rather than independent metabolic risk factors in the respective groups. In contrast, adiponectin concentrations associated strongly and favorably with all recorded lipid variables except for total and LDL cholesterol as reported in the population at large [1, 2, 5, 7], but consistently with high systolic and diastolic as well as mean blood pressure in RA subjects. The latter relationships in RA persisted even after additional potential determinants of hypertension including lifestyle factors and leflunomide use were accounted for. Our finding that adiponectin concentration-metabolic risk factor relationships differed by RA status suggests that the influence of adiponectin on metabolic cardiovascular risk factors as identified in non-RA subjects cannot be merely extrapolated to patients with RA. 

 The cIMT was similar in RA compared with non-RA subjects. Hence, the relative potential adverse influence of circulating adiponectin on blood pressure may be counterbalanced by its beneficial impact on lipid metabolism, thereby resulting in an overall neutral effect on atherosclerosis in RA. Taken together, our results suggest that interventions that alter the production or inhibit the effects of adiponectin may influence individual metabolic risk factors but not overall adiponectin-mediated cardiovascular risk and disease in RA. Interestingly, glucocorticoid [50] and synthetic and biologic DMARD [51–53] can also alter adiponectin production in RA. Leptin inhibition would not be expected to influence either leptin-mediated metabolic risk or atherosclerosis in RA. 

 Previous studies reported higher or similar serum leptin and adiponectin concentrations in RA compared to non-RA subjects [1, 10–19]. In the present study, we found that RA is associated with reduced circulating concentrations of both adipokines. Congruent with this finding and of likely importance in the present context, Ukkola and colleagues recently reported disparities in body composition, the insulin response to glucose and plasma lipid concentrations according to the different alleles of the gene encoding adiponectin in black and white subjects [34]. Reported findings and our results therefore strongly suggest that adipokine production in RA and the influence of circulating adipokines on metabolic cardiovascular risk factors are population specific and, hence, also argue against extrapolation of findings on adipokine metabolism from one population to another in cardiovascular risk management. 

 Hypertension is associated with reduced adiponectin concentrations, and low adiponectin levels were shown to increase the risk of hypertension in the population at large [5, 54, 55]. The strong and independent association of adiponectin concentrations with blood pressure in our patients with RA is therefore unexpected. We included only black Africans, whereas the impact of adiposity on cardiovascular risk may differ by population grouping [35]. Indeed, in a previous investigation in 33 white patients with RA that were treated with the tumor necrosis factor-alpha antagonist infliximab, we found that adiponectin concentrations were inversely related to atherogenic lipid ratios and plasma glucose concentrations but not to blood pressure [56]. 

 Our cross-sectional study design precludes, however, drawing inferences on the direction of causality. Thus, our findings on adiponectin-blood pressure relationships in RA could conceptually also have resulted from a compensatory increase of adiponectin production in response to refractory hypertension, as observed in our patients, and caused by factors other than adiponectin, and in an attempt to reduce blood pressure values. A compensatory increase in adiponectin production in the presence of high grade inflammation and in an attempt to reduce inflammation was previously also postulated to underlie increased adiponectin serum concentrations in patients with RA from developed populations [10, 11]. Further prospective longitudinal and mechanistic studies are required to elucidate the relationship between circulating adiponectin concentrations and blood pressure in RA. Also, whether the positive adiponectin concentration-blood pressure relationships in RA as found in the present investigation translate in accelerated incident cardiovascular risk and disease merits additional investigation. 

 The present study has further limitations. Carotid artery plaques are more strongly associated with CAD and lipids than cIMT that relates more closely to stroke and blood pressure [35]. Nevertheless, both cIMT and plaque predict future cardiovascular event rates in RA and non-RA subjects irrespective of population grouping [57–60]. We measured circulating total adiponectin concentrations. Amongst its different isoforms, it is high molecular weight adiponectin that reportedly confers the vascular-protective activities in the general population [61]. 

 In conclusion, considering previously reported findings, this study suggests that altered adipokine production in RA is population specific. RA modifies adiponectin concentration-metabolic risk factor relationships. Individual cardiovascular risk factors and particularly serum lipid concentrations require close monitoring upon employing interventions that alter adiponectin production or inhibit its effects in RA. However, whereas leptin and adiponectin inhibition could improve disease activity, this intervention may also not result in altered overall cardiovascular and disease in RA.

## Figures and Tables

**Table 1:** Baseline characteristics in subjects with and without rheumatoid arthritis.

Characteristic	Rheumatoid arthritis		*P*
	Present (*n* = 119)	Absent (*n* = 158)	
Demographics			
Age	55.8 (10.2)	56.5 (10.9)	0.6
Female (%)	89.1	86.1	0.4
Lifestyle factors			
Alcohol use (%)	0.8	17.7	0.006
Units per week, n*	0.01 (0.07)	0.27 (1.09)	0.001
Exercise (%)	40.3	43.0	0.6
Hours per week, n*	0.01 (1.00)	1.7 (2.4)	0.0001
Smoking (%)	3.4	8.2	0.1
Cigarettes per day	0.05 (0.37)	0.15 (0.69)	0.06
Anthropometric measures			
BMI, kg/m^2^	29.3 (6.6)	33.7 (8.0)	<0.0001
Waist circumference, cm	93.3 (13.4)	97.5 (15.1)	0.02
Waist-hip ratio*	0.86 (0.12)	0.85 (0.10)	0.4
Cardiovascular drugs			
Antihypertensive agents			
Use (%)	54.6	40.0	0.02
Number	1.0 (1.1)	0.5 (0.7)	<0.0001
Diuretic (%)	39.5	38.6	0.9
ACEI (%)	39.5	7.0	<0.0001
CCB (%)	17.7	5.1	0.002
BB (%)	3.4	0	—
ARB (%)	0.8	0	—
Glucose lowering agents			
Oral glucose lowering agent (%)	13.5	10.1	0.4
Insulin (%)	0.8	2.5	0.6
Statin (%)	19.4	0	—
NSAID (%)	6.7	4.4	0.3
Systemic inflammation			
CRP (mg/L)*	7.0 (3.1)	6.7 (3.1)	0.7

Results are expressed as mean (SD) or proportions/percentages. *N*: number; BMI: body mass index; ACEI: angiotensin converting enzyme inhibitor; CCB: calcium channel blocker, BB: beta blocker, ARB: angiotensin receptor blocker; NSAID: nonsteroidal anti-inflammatory agent; CRP: C-reactive protein.

*Nonnormally distributed variables for which geometric mean (SD) is given.

^†^Denotes active RA.

**Table 2:** Metabolic cardiovascular risk factor profiles and carotid atherosclerosis in subjects with and without rheumatoid arthritis.

Characteristic	Rheumatoid arthritis		
	Present (*n* = 119)	Absent (*n* = 158)	
Categorical variables			OR (95% CI)*

Hypertension (%)	74.0	65.2	1.47 (0.87 to 2.48)
Total C-HDL C ratio > 4	**21.7**	**33.8**	**0.53 (0.29 to 0.96)**
Diabetes (%)	16.0	12.0	1.46 (0.74 to 2.88)

Continuous variables			P*

Blood pressure values			
SBP, mmHg	140 (25)	137 (22)	0.3
DBP, mmHg	86 (15)	87 (13)	0.5
MBP, mmHg	104 (17)	104 (15)	0.9
Lipid values			
Total C, mmol/L	**4.7 (0.9)**	**5.1 (1.2)**	**0.02**
HDL C^†^, mmol/L	**1.48 (1.34)**	**1.39 (1.32)**	**0.04**
Total C-HDL C ratio	**3.2 (1.1)**	**3.7 (1.3)**	**0.0002**
LDL C, mmol/L	**2.6 (0.8)**	**3.0 (1.0)**	**0.003**
Non HDL C, mmol/L	**3.1 (0.9)**	**3.6 (1.2)**	**0.0001**
Trig^†^, mmol/L	**1.1 (1.7)**	**1.2 (1.6)**	**0.03**
Trig-HDL C ratio^†^	**0.73 (2.05)**	**0.87 (1.85)**	**0.01**
Cigarettes smoked per day^†^, *n*	0.05 (0.37)	0.15 (0.68)	0.08
Glucose^†^, mmol/L	5.3 (1.4)	5.5 (1.4)	0.1
Carotid atherosclerosis			
cIMT, mm	0.694 (0.098)	0.704 (0.121)	0.5

Results are expressed in mean (SD) or proportions/percentages. Significant associations are shown in bold. C: cholesterol; HDL: high density lipoprotein; Trig: triglycerides; SBP: systolic blood pressure; DBP: diastolic blood pressure; MBP: mean blood pressure; LDL: low density lipoprotein; *n*: number; CHD: coronary heart disease; CVD: cardiovascular disease; cIMT: carotid intima-media thickness.

*Adjusted for age and sex with additional adjustment for antihypertensive, statin, and glucose lowering therapy in models that included blood pressure, lipid, and glucose variables, respectively.

^†^Nonnormally distributed variables for which geometric means (SD) are given.

**Table 3:** Impact of RA on the age and sex adjusted relationships between serum adipokine concentrations and metabolic risk factors and carotid atherosclerosis.

	Leptin*					Adiponectin*				
Potential characteristic	Interaction *P*	RA (*n* = 112)		Non-RA (*n* = 158)		Interaction *P*	RA (*n* = 119)		Non-RA (*n* = 77)	
		Partial *R*	*P*	Partial *R*	*P*		Partial *R*	*P*	Partial *R*	*P*
SBP	0.6	0.110	0.3	0.019	0.8	0.0009	0.257	0.005	0.093	0.4
DBP	0.0008	0.248	0.009	0.044	0.6	<0.0001	0.174	0.06	−0.009	0.9
MBP	0.09	0.201	0.04	0.034	0.7	<0.0001	0.229	0.01	0.037	0.8
Total C	0.0003	−0.022	0.8	0.123	0.1	0.2	−0.113	0.2	0.040	0.7
HDL C*	0.9	−0.117	0.2	−0.089	0.3	<0.0001	0.442	<0.0001	0.224	0.06
Total C-HDL C ratio	<0.0001	0.095	0.3	0.178	0.03	<0.0001	−0.466	<0.0001	−0.202	0.08
LDL C	<0.0001	−0.014	0.9	0.148	0.07	0.06	−0.187	0.049	−0.069	0.6
Non HDL C	0.002	0.048	0.6	0.159	0.05	0.0008	−0.322	0.0005	−0.021	0.9
Trig*	1.0	0.124	0.2	0.161	0.046	0.8	−0.366	<0.0001	−0.295	0.01
Trig-HDL C ratio*	0.9	0.139	0.2	0.163	0.045	0.0009	−0.456	<0.0001	−0.347	0.002
Glucose*	0.0003	0.084	0.4	0.082	0.3	0.1	−0.142	0.1	−0.152	0.2
cIMT	0.006	0.144	0.1	0.112	0.3	0.2	−0.076	0.4	−0.008	1.0

Additional adjustment was made for antihypertensive agent use, statins and oral glucose lowering agent, and insulin use in models that included blood pressure, lipid variables, and glucose concentrations, respectively. SBP: systolic blood pressure; DBP: diastolic blood pressure; C: cholesterol; HDL: high density lipoprotein; LDL: low density lipoprotein; trig: triglycerides; CRP: C-reactive protein; cIMT: carotid intima-media thickness.

*Logarithmically transformed in view of nonnormal distribution.

**Table 4:** Impact of RA on the independent relationships of serum leptin and adiponectin concentrations with metabolic risk factors and carotid atherosclerosis.

	Leptin*				Adiponectin*			
Potential characteristic	RA (*n* = 112)		Non-RA (*n* = 158)		RA (*n* = 119)		Non-RA (*n* = 77)	
	Partial *R*	*P*	Partial *R*	*P*	Partial *R*	*P*	Partial *R*	*P*
SBP	−0.048	0.6	−0.018	0.9	0.331	0.0006	0.067	0.7
DBP	0.109	0.3	0.014	0.9	0.280	0.004	−0.050	0.8
MBP	0.042	0.7	−0.000	1.0	0.329	0.0007	0.001	1.0
Total C	−0.035	0.7	0.091	0.3	−0.115	0.3	−0.018	0.9
HDL C*	−0.113	0.3	0.009	0.9	0.335	0.0005	0.103	0.6
Total C-HDL C ratio	−0.076	0.5	0.068	0.5	−0.363	0.0002	−0.120	0.5
LDL C	−0.024	0.8	0.064	0.5	−0.149	0.1	−0.039	0.8
Non HDL C	0.038	0.7	0.087	0.4	−0.263	0.007	−0.036	0.8
Trig*	0.120	0.2	0.093	0.3	−0.271	0.005	−0.281	0.1
Trig-HDL C ratio*	0.136	0.2	0.066	0.5	−0.347	0.0003	−0.291	0.1
Glucose*	0.012	0.9	−0.020	0.8	−0.125	0.2	−0.151	0.4
cIMT	0.123	0.2	0.023	0.9	−0.096	0.3	0.008	1.0

Age, sex, body mass index (for leptin), waist (for adiponectin), C-reactive protein concentrations, angiotensin converting enzyme inhibitors and diuretic use, and stain therapy were adjusted for in each model. Additional adjustment was made for the use of any antihypertensive agents, oral glucose lowering agent and insulin use in models that included blood pressure and glucose concentrations, respectively. SBP: systolic blood pressure; DBP: diastolic blood pressure; C: cholesterol; HDL: high density lipoprotein; LDL: low density lipoprotein; trig: triglycerides; CRP: C-reactive protein; cIMT: carotid intima-media thickness.

*Logarithmically transformed in view of nonnormal distribution.
